# Highly pleiotropic variants of human traits are enriched in genomic regions with strong background selection

**DOI:** 10.1007/s00439-021-02308-w

**Published:** 2021-07-06

**Authors:** Irene Novo, Eugenio López-Cortegano, Armando Caballero

**Affiliations:** 1 Centro de Investigación Mariña, Universidade de Vigo, Facultade de Bioloxía, 36310 Vigo, Spain; 2 Present Address: Institute of Evolutionary Biology, School of Biological Sciences, University of Edinburgh, Edinburgh, EH9 3FL UK

## Abstract

Recent studies have shown the ubiquity of pleiotropy for variants affecting human complex traits. These studies also show that rare variants tend to be less pleiotropic than common ones, suggesting that purifying natural selection acts against highly pleiotropic variants of large effect. Here, we investigate the mean frequency, effect size and recombination rate associated with pleiotropic variants, and focus particularly on whether highly pleiotropic variants are enriched in regions with putative strong background selection. We evaluate variants for 41 human traits using data from the NHGRI-EBI GWAS Catalog, as well as data from other three studies. Our results show that variants involving a higher degree of pleiotropy tend to be more common, have larger mean effect sizes, and contribute more to heritability than variants with a lower degree of pleiotropy. This is consistent with the fact that variants of large effect and frequency are more likely detected by GWAS. Using data from four different studies, we also show that more pleiotropic variants are enriched in genome regions with stronger background selection than less pleiotropic variants, suggesting that highly pleiotropic variants are subjected to strong purifying selection. From the above results, we hypothesized that a number of highly pleiotropic variants of low effect/frequency may pass undetected by GWAS.

**Supplementary Information:**

The online version contains supplementary material available at 10.1007/s00439-021-02308-w.

## Introduction

The analyses of thousands of genetic variants obtained in the last decades by Genome-Wide Association Studies (GWAS) have provided a great advance in the knowledge of the understanding of genetic variation, particularly for human traits (Visscher et al. 2017). One issue arising from these analyses is the ubiquity of pleiotropy, i.e., the observation that a genetic variant may affect more than one trait (Wright 1968; Kacser and Burns 1981; Stearns 2010; Paaby and Rockman 2013). Several recent studies have shown that a great proportion of the human genome is involved in pleiotropic effects (e.g., Wang et al. 2010; Sivakumaran et al. 2011; Pickrell et al. 2016; Chesmore et al. 2018; Jordan et al. 2019; Watanabe et al. 2019; Shikov et al. 2020) and it has been suggested that complex traits are driven by an enormously large number of genes, implying that pleiotropy is the rule rather than the exception (Boyle et al. 2017). The latest meta-analysis on pleiotropic variants carried out by Shikov et al. (2020), and based on more than 500 complex traits, concludes that about 180 Mbs of the human genome are covered by pleiotropic loci and about 50% of SNPs are associated with more than one phenotype. Another recent study (Watanabe et al. 2019) suggests that this proportion is even larger (60%). Highly pleiotropic variants are generally associated with broadly expressed genes with ubiquitous functions, such as matrisome components, developmental and immunological system genes, and growth cell regulators (Shikov et al. 2020).

An observation made by Shikov et al. (2020) is that rare variants tend to be less pleiotropic than common ones. This result is coherent with the observation that natural selection against deleterious mutations has been shown to operate on complex trait variation (Gazal et al. 2018; Zeng et al. 2018). Thus, if pleiotropic variants affecting human diseases tend to be deleterious, highly pleiotropic variants would be expected to be removed from the population or kept at low frequencies (Paaby and Rockman 2013). Shikov et al. (2020) also showed that more pleiotropic variants have higher gene expression than less pleiotropic ones, although they did not compare the mean effect sizes of variants across different degrees of pleiotropy. A previous analysis of pleiotropy of human genes showed, however, a tendency for more pleiotropic variants to have larger effect sizes than less pleiotropic ones (Chesmore et al. 2018), an observation also made for other species (Wagner and Zhang 2011). Since the detection power of GWAS increases with the frequency and effect size of variants (Hill and Zhang 2012; Visscher et al. 2017), it would be expected that highly pleiotropic variants found by GWAS would tend to have substantial effect sizes and frequencies, explaining the latter results. Nevertheless, the observation of a higher frequency and also a higher effect size for highly pleiotropic variants seems to be contradictory with the hypothesis that highly pleiotropic loci are strongly affected by purifying selection. A way to ascertain the support for the purifying selection hypothesis is to investigate the degree of background selection associated with loci with different degrees of pleiotropy. This can be done by examining the mean value of the *B* statistic (McVicker et al. 2009) ascribed to the genomic regions where variants with different degrees of pleiotropy are allocated. The *B* statistic indicates the expected fraction of neutral diversity that remains at a given genomic site because of the effect of background selection (Charlesworth and Charlesworth 2010, Chap. 8). Under the purifying selection hypothesis, and for a constant recombination rate in a given genomic region, it would be expected that more pleiotropic variants in that region were associated with lower values of the *B* statistic than less pleiotropic ones, implying a larger effect of negative selection.

Here, we carried out an analysis of variants recovered from the GWAS Catalog for 41 human traits and diseases to investigate the mean frequency, effect size, recombination rate and intensity of background selection associated with variants with different degrees of pleiotropy. In addition, we investigated the intensity of background selection associated with the datasets of pleiotropic variants analyzed by Pickrell et al. (2016), Watanabe et al. (2019) and Shikov et al. (2020). Overall, the results suggest that more pleiotropic variants are located in regions with stronger background selection.

## Methods

The analyses first reported in this paper were carried out on the NHGRI-EBI GWAS Catalog data (MacArthur et al. 2017), previously analyzed by López-Cortegano and Caballero (2019) for a different purpose. Briefly, the GWAS Catalog was processed by filtering incomplete or low informative data and by clustering together traits with a highly overlapping genetic background. All data manipulation, including statistical analyses, was carried out using the R language (R Core Team 2017).

We considered SNPs for which information on the mapped gene, the effect, reported as an odds ratio or beta-coefficient, the frequency of the risk allele, and the reported *p* value, were available in the Catalog. For odds ratio traits, the corresponding variant effects for liability were estimated by the method of So et al. (2011). We limited our study to the most significant associations, disregarding SNPs with a significance level higher than the standard *p* = 5 × 10^−8^. Only one SNP per associated Catalog gene (that with the lowest *p* value) was considered, and the corresponding gene or intergenic name associated with that SNP was assumed to be a potential causal locus. The contribution to heritability from each locus was calculated as *h*^2^ = 2*β*^2^*q*(1 − *q*) where *β* is the locus estimated effect and *q* its frequency. For the sake of robustness, only traits with a wide and well-known genetic background composed by at least 30 unique genes detected were considered. In addition, we restricted the traits analyzed to those represented by at least three different studies. More details of the procedure can be found in López-Cortegano and Caballero (2019). In total, the dataset analyzed was composed of autosomal loci corresponding to 41 human traits which can be classified in 10 functional domains (Supplemental Table S1).

The detected SNPs and associated loci were classified as pleiotropic of degree 1, 2, 3, etc. if they were associated with 1 (non-pleiotropic), 2, 3, etc. traits. The average homozygous effect size, minor allele frequency (MAF) and contribution to heritability from each locus, were obtained for each pleiotropy degree.

The value of the *B* statistic attached to each genomic position of the genome represents the expected reduction in nucleotide diversity at a neutral site due to purifying selection at other sites (McVicker et al. 2009). These authors made a systematic search for signatures of selection by analyzing the genomic distribution of human polymorphisms and sequence differences with other primate species. By applying a theoretical model of background selection (Hudson and Kaplan 1995; Nordborg et al. 1996) to conserved and neutral regions, they could calculate the value of this statistic along the human genome. A value of *B* = 1 indicates that no neutral diversity has been lost by selection, whereas a value of zero would indicate a maximal loss because of purifying selection. A reduction in neutral diversity for a given genomic region is a function of the intensity of purifying selection and the rate of recombination, as the impact of selection on diversity is higher in low recombination regions (Charlesworth et al. 1993; Santiago and Caballero 1998). The average *B* value across the autosomal genome is of about 0.74–0.81 (McVicker et al. 2009).

We investigated the relationship between the degree of pleiotropy and the mean intensity of background selection in our own data and in that obtained by Pickrell et al. (2016), Watanabe et al. (2019) and Shikov et al. (2020). Pickrell et al. (2016) studied 42 human traits (using GWAS from different studies and their own one) and identified 348 genomic regions with SNPs associated with more than 1 trait (available from their Supplementary Table 1). Watanabe et al. (2019) studied 236,638 SNPs from the UK Biobank (their Supplementary Table 12), 11,544 genes (their Supplementary Table 7) and 3,362 loci groups (of physically overlapping loci; their Supplementary Table 4) associated with 558 traits (grouped in 24 domains). Finally, using the UK Biobank data, Shikov et al. (2020) were able to identify 149,345 pleiotropic SNPs from which 64,545 were regarded as high-confidence biologically pleiotropic variants (their Additional Data 5). The pleiotropic variants were located in 1314 genomic regions along the human genome (their Table S1), encompassing about 180 Mbs. These genomic regions were classified according to the median or maximal degree of pleiotropy of the variants encompassed within them.

We analyzed the relationship between the strength of background selection and the degree of pleiotropy at the level of genomic regions, genes or SNPs associated with them using the coefficient of simple linear regression (*b*) for the value of *B* on the degree of pleiotropy. Since, as mentioned above, the value of *B* depends both on the intensity of natural selection and the rate of recombination (RR), we also obtained the partial regression coefficients (*b′*) of *B* on the degree of pleiotropy and RR. These were obtained with the *R* command summary (lm(*y* ~ *x*_1_ + *x*_2_)), where *y* is the dependent variable (*B*) and *x*_1_ and *x*_2_ are the predictor variables (degree of pleiotropy and RR, respectively). For genomic regions we averaged the *B* and RR values for all positions within each region. For genes, we averaged the corresponding values for all positions from the start to the end of the gene. Finally, for SNPs, the values of *B* for each SNP position were considered. All genomic regions, gene and SNP coordinates were fitted to the genome version GRCh37 (hg19), using the dbSNP database (Sherry et al. 2001) (ftp://ftp.ncbi.nlm.nih.gov/snp/organisms/archive/human_9606_b144_GRCh37p13/VCF) for SNPs, and the RefSeq database (O’Leary et al. 2016) (ftp://ftp.ncbi.nlm.nih.gov/refseq/H_sapiens/annotation/GRCh37_latest/refseq_identifiers/GRCh37_latest_genomic.gff.gz) for genes. Recombination rates for each SNP, gene or genomic region fitted to the GRCh37 coordinates were obtained from the human genetic map (Myers et al. 2005). Since many variants are detected in the MHC region, which is not representative of the rest of the genome in terms of recombination rate or *B* statistic due to its high diversity and linkage disequilibrium (Traherne 2008), for this analysis, we discarded the SNPs, genes and genomic regions located in, or strongly linked to that region, removing data from 25 to 34 Mb of chromosome 6.

## Results

**Fig. 1:**
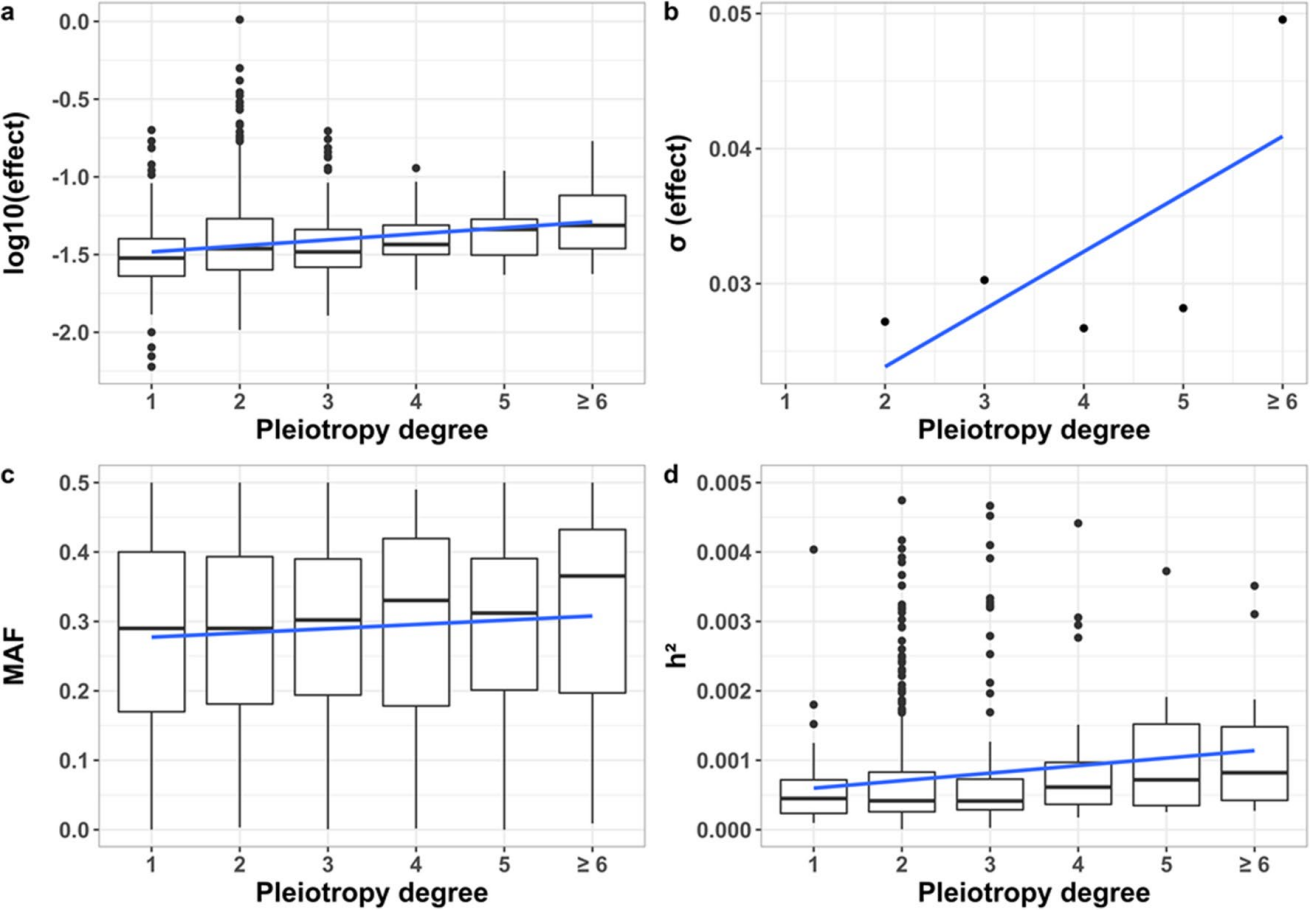
**a** Relationship between the estimated effect of variants and the degree of pleiotropy (*b* = 0.035, *R*^2^ = 0.07, *F* = 110.6, *p* < 2 × 10^−16^). **b** Relationship between the standard deviation of the effect sizes of pleiotropic variants and the degree of pleiotropy (*b* = 0.008, *R*^2^ = 0.35, *F* = 5.78, *p* = 0.04). **c** Relationship between the minor allele frequency (MAF) of SNPs and the degree of pleiotropy (*b* = 0.006, *R*^2^ = 0.0002, *F* = 14.38, *p* = 0.0002). **d** Relationship between the heritability contributed by the variants (*h*^2^) and the degree of pleiotropy (*b* = 3.15 × 10^−4^, *R*^2^ = 0.009, *F* = 10.16, *p* = 0.001). Simple regression lines are shown

The total number of pleiotropic loci found in our study was 629, which is a 23% of all loci analyzed (Table S1). Gastrointestinal, skeletal and cardiovascular functional domains presented the highest proportions of pleiotropic loci when averaging traits (62, 61 and 60%, respectively), and the neurological/psychiatric domain, the lowest one (18%) (Supplementary Figure S1). As expected, the higher the pleiotropy degree, the lower the number of variants found, with the highest degree being 12 (Supplementary Fig. S2). The mean effect size steadily increased with the pleiotropy degree (Fig. 1a, regression coefficient *b* = 0.035, *p* < 2 × 10^−16^), and the same was observed for the standard deviation of effect sizes (Fig. 1b) (*b* = 0.008, *p* = 0.04). The MAF of variants gradually increased with the pleiotropy degree (Fig. 1c, *b* = 0.006, *p* < 2 × 10^−4^), and this, along with the increased effects sizes, accounted for a higher contribution to heritability for the most pleiotropic classes (Fig. 1d, *b* = 3.15 × 10^−4^, *p* = 0.001).

**Fig. 2:**
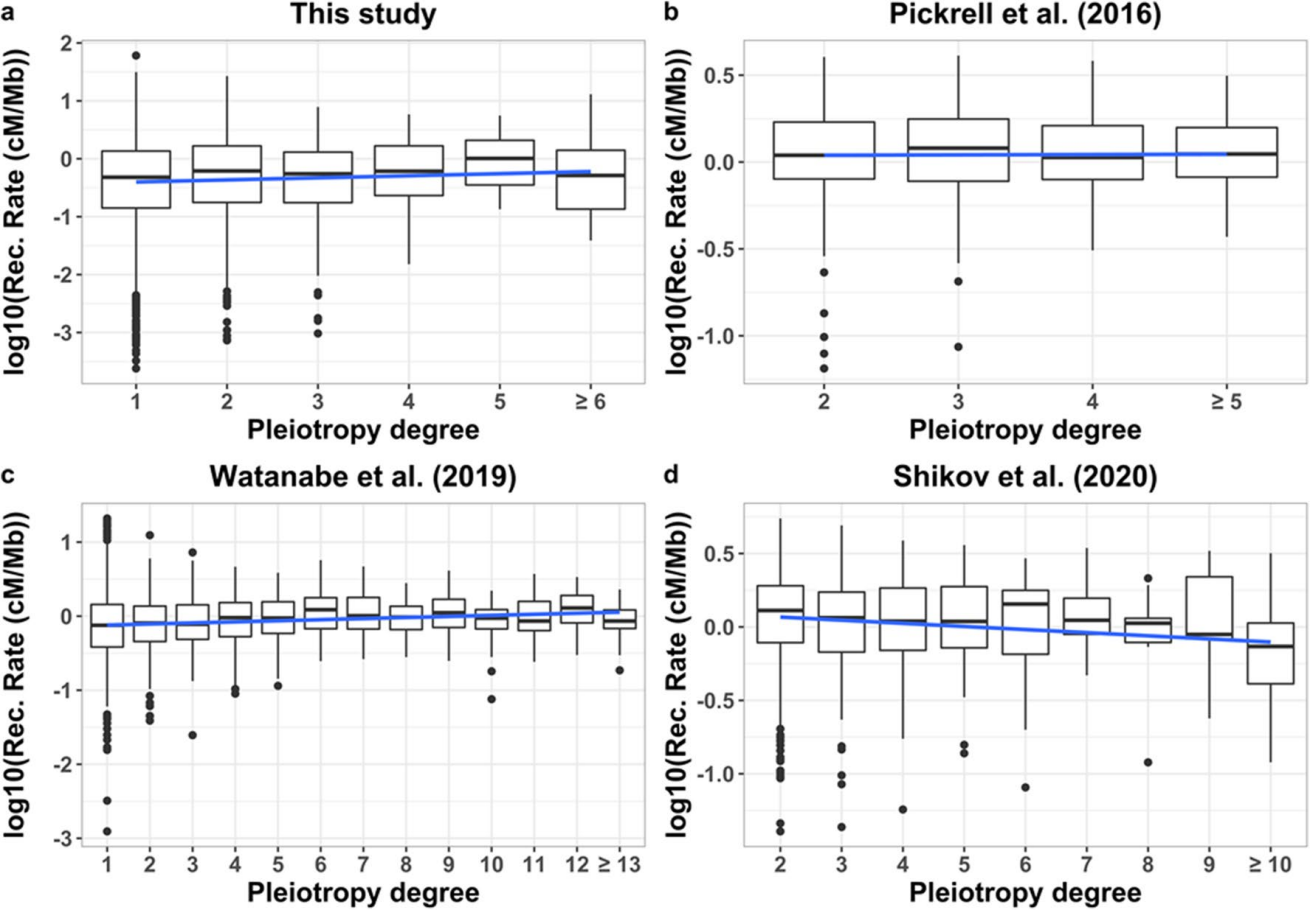
Relationship between the recombination rate (RR in log_10_[cM/Mb]) of each variant genomic position and the degree of pleiotropy. **a** Data from the dataset corresponding to Fig. 1 considering the average RR of genes (*b* = 0.032, *R*^2^ = 0.0009, *F* = 3.57, *p* = 0.06). **b** Data from Pickrell et al. (2016) considering the average RR of genomic regions (*b* = − 0.004, *R*^2^ = 0.0006, *F* = 0.19, *p* = 0.7). **c** Data from Watanabe et al. (2019) considering the average *RR* of genomic regions and the degree of pleiotropy of domains (*b* = 0.014, *R*^2^ = 0.010, *F* = 34.65, *p* = 4 × 10^−9^). **d** Data from Shikov et al. (2020) considering the average *RR* of genomic regions and the maximal degree of pleiotropy of domains (*b* = − 0.017, *R*^2^ = 0.01, *F* = 17.4, *p* = 3 × 10^−5^). Simple regression lines are shown

The rate of recombination was almost invariable across the different degrees of pleiotropy, with a tendency to be positively correlated with the pleiotropy level, and only slightly negative for the data of Shikov et al. (2020) (Fig. 2).

**Fig. 3:**
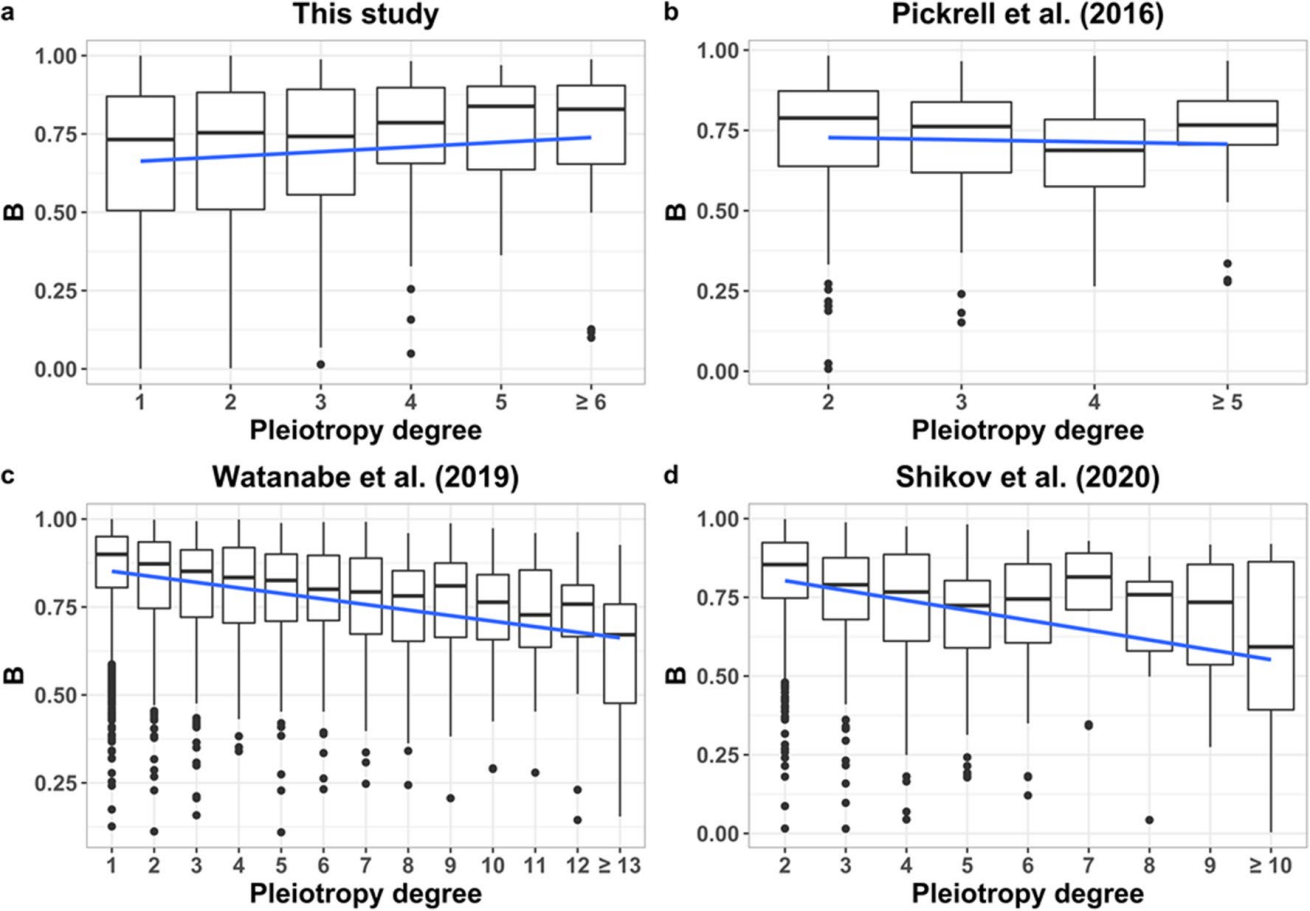
Relationship between the average background selection statistic (*B*) of each variant genomic position and the degree of pleiotropy. **a** Data from the dataset corresponding to Fig. 1 considering the average *B* value of genes (simple regression *b* = 0.015, *R*^2^ = 0.002, *F* = 6.25, *p* = 0.01; partial regression *b*′ = 0.010, *R*^2^ = 0.22, *F* = 362.7, *p* = 0.06). **b** Data from Pickrell et al. (2016) considering the average *B* value of genomic regions (simple regression *b* = − 0.009, *R*^2^ = 0.0002, *F* = 1.58, *p* = 0.2; partial regression *b*′ = − 0.007, *R*^2^ = 0.40, *F* = 109.6, *p* = 0.2). **c** Data from Watanabe et al. (2019) considering the average *B* value of genomic regions and the degree of pleiotropy of domains (simple regression *b* = − 0.015, *R*^2^ = 0.09, *F* = 327.7, *p* < 2 × 10^−16^; partial regression *b*′ = − 0.018, *R*^2^ = 0.24, *F* = 534, *p* < 2 × 10^−16^). **d** Data from Shikov et al. (2020) considering the average *B* value of genomic regions and the maximal degree of pleiotropy of domains (simple regression *b* = − 0.026, *R*^2^ = 0.06, *F* = 87.02, *p* < 2 × 10^−16^; partial regression *b*′ = − 0.022, *R*^2^ = 0.38, *F* = 400.5, *p* < 2 × 10^−16^). The partial regression coefficients of the value of *B* on the rate of recombination are *b*′ = 0.158, 0.423, 0.151 and 0.332 for the four datasets of **a**–**d**, respectively, all of them significant with *p* < 2 × 10^−16^. Simple regression lines are shown

The relationship between the degree of pleiotropy and the strength of background selection (*B* statistic) is given in Fig. 3. The relationship was non-significant for our own data (Fig. 3a, partial regression of *B* on the degree of pleiotropy of *b*′ = 0.010, *p* = 0.06) and for the data of Pickrell et al. (2016) (Fig. 3b, *b*′ = − 0.007, *p* = 0.2). However, for the two much larger datasets of Watanabe et al. (2019) (Fig. 3c, *b*′ = − 0.018, *p* < 2 × 10^−16^) and Shikov et al. (2020) (Fig. 3d, *b*′ = − 0.022, *p* < 2 × 10^−16^), the relationship was significantly negative and of similar magnitude. The results presented in Fig. 3 excluded MHC regions, however, when these were considered the results were similar, with partial regression of *B* on the degree of pleiotropy of: *b*′ = 0.012 (*p* = 0.02), − 0.004 (*p* = 0.4), − 0.017 (*p* < 2 × 10^−16^), − 0.013 (*p* < 2 × 10^−16^), respectively. The results of Watanabe et al. (2019) in Fig. 3c refer to the average *B* value of genomic regions considering domains, but the results were similar if traits (rather than domains) or genes or SNPs were considered instead (Fig. S3). Analogously, the results of Shikov et al. (2020) in Fig. 3d refer to genomic regions regarding their maximal pleiotropic degree, but similar results were obtained when median pleiotropic degrees of each region were assumed, or if SNPs were considered instead (Fig. S4).

## Discussion

The results from our data show that about 23% of variants associated with 41 diseases and other human traits are pleiotropic, and that variants with higher degree of pleiotropy are more common and have average larger effect sizes than less pleiotropic or non-pleiotropic variants (Fig. 1). The proportion of pleiotropic loci found is lower than that reported by Chesmore et al. (2018) (44%) and by Shikov et al. (2020) (49%), and much smaller than that reported by Watanabe et al. (2019) (60%). These differences, however, can be ascribed to a much lower number of traits considered in our study (41) with respect to those considered by Chesmore et al. (2018) (1094 traits), Watanabe et al. (2019) (558 traits) and by Shikov et al. (2020) (543 traits). In addition, as suggested by Shikov et al. (2020), the large proportion of pleiotropic variants detected by Watanabe et al. (2019) could be explained by the use by these authors of sparsely defined trait domains.

In agreement with the results of Chesmore et al. (2018), we found a tendency for the average mean effect size of pleiotropic loci to increase with the degree of pleiotropy (Fig. 1a), which is also in agreement with other observations (Wagner and Zhang 2011). However, Chesmore et al. (2018) reported a decrease in the variance of effect sizes with the degree of pleiotropy whereas we observed an increase in the standard deviation (Fig. 1b). The discrepancy is due to a different way of calculation. Chesmore et al. (2018) calculated the variance of the average values of the multiple effects ascribed to a pleiotropic locus. As they discussed, because the larger the degree of pleiotropy the larger the number of effect sizes averaged, the variance of the mean is decreased with the degree of pleiotropy because of the law of large numbers. In fact, doing the calculation of the variance in that way, we also obtained a decline in the standard deviation of effects within the degree of pleiotropy (Supplemental Fig. S5). In contrast, in our Fig. 1b, we obtained the standard deviation of effect sizes within pleiotropic loci, and then averaged those standard deviations over loci with the same pleiotropic class, observing an increase in the standard deviation with the degree of pleiotropy. Therefore, more pleiotropic loci have a higher disparity of effects on the multiple traits they affect than less pleiotropic loci.

Another difference between our results and those of Chesmore et al. (2018) refers to the levels of pleiotropy found. Whereas we found loci with a maximum of 12 (dichotomous and quantitative traits) associated traits, Chesmore et al. (2018) investigated only dichotomous traits and found loci with a degree of pleiotropy up to 53. This difference can be again ascribed to the much larger number of traits considered by Chesmore et al. (2018) (more than 1000 versus 41). To have the highest possible robustness in the data, we grouped traits with similar genetic architecture, and we analyzed a very restricted set of traits, in particular, only traits for which at least three studies had been reported in the Catalog and for which at least 30 loci had been detected.

We found an increase in minor allele frequency with the degree of pleiotropy (Fig. 1c), in accordance with the observation of Shikov et al. (2020) that rare variants tend to be less pleiotropic than common ones. In agreement with this increase in frequency and effect sizes, the proportional contribution to heritability for each of the traits from more pleiotropic loci was found to be higher than that of less pleiotropic or non-pleiotropic ones (Fig. 1d). Thus, it appears that highly pleiotropic loci may contribute substantially to heritability. This observation is concordant with the idea of the ‘omnigenic’ model suggested by Boyle et al. (2017), for which most loci of the genome might contribute in one way or another to heritability, with genes of high effect size (possibly the most pleiotropic ones) at the center of the genomic network. To explain the larger frequency for more pleiotropic variants, Shikov et al. (2020) provided three possible explanations. First, that a lack of rare pleiotropic variants may be a consequence of a lack of statistical power for their detection. Second, that common variants may have spurious pleiotropy resulting from linkage disequilibrium with different causal variants. In fact, inferring pleiotropy from molecular markers is difficult if the linkage disequilibrium relationships between markers and causal variants are not known with precision (Gianola et al. 2015). And third, that natural purifying selection against highly pleiotropic deleterious variants of large effect size would result in segregating pleiotropic variants with lower effect sizes and higher frequencies. As stated by Shikov et al. (2020), the fact that natural selection against deleterious mutations has been shown to operate on complex trait variation (Gazal et al. 2018; Zeng et al. 2018), would support the third explanation. However, the other two explanations may also play a role.

We analyzed the relationship between the degree of pleiotropy of variants and the strength of background selection attached to their positions. We found that, for the analysis with fewer traits (Pickrell et al. 2016, and our own study) with about 40 traits each, there was a non-significant relationship between *B* and the degree of pleiotropy (Fig. 3a, b). Nevertheless, some of the most pleiotropic loci found in our study (Table S2) were associated with low values of *B*, particularly gene GCKR (*B* = 0.099), which was also found as highly pleiotropic by Chesmore et al. (2018), thus denoting a high impact of background selection. For the larger datasets (Watanabe et al. 2019; Shikov et al. 2020) with many more traits (more than 500) and pleiotropic SNPs (about one hundred and fifty thousand), there was a consistent significant and negative relationship between *B* and the degree of pleiotropy (Fig. 3c, d). The discrepancy between the non-significant relationships found for the two first datasets and these ones can be that the latter are more comprehensive studies, but there may be other explanations. The results in Fig. 3 refer to different sources of data, considering the average *B* value of genes in the case of our own data, and that of genomic regions in the case of the other studies. However, for Watanabe et al. (2019) data, the trends were repeated when the average *B* was obtained from genes (Fig. S3c, d) as well as for individual SNPs (Fig. S3e, f). Moreover, for Shikov et al. (2020) results, the trends were also similar if individual SNPs were considered (Fig. S4c, d). These general tendencies are also shown in Supplemental Material Fig. S6, which shows the mean value of *B* for a range of pleiotropic degree classes for the main datasets available. Note that the data from Pickrell et al. (2016) and Shikov et al. (2020) do not have results for the non-pleiotropic class, which could contribute to the non-significant relationship found in the former. Therefore, the negative relationship found between *B* and the degree of pleiotropy is very robust. An additional source of difference between the datasets is that our results arise from the GWAS Catalog whereas those from Watanabe et al. (2019) and Shikov et al. (2020) were obtained from the UK Biobank, and there could be differences between both sources of data, which remain to be disclosed.

Since the relationship between the rate of recombination and the degree of pleiotropy was nearly invariable (Fig. 2), the negative relationship between *B* and the degree of pleiotropy indicates that the reduction of *B* with the degree of pleiotropy is not explained by a reduced recombination rate for highly pleiotropic regions. In any case, we obtained the partial regression of *B* on the degree of pleiotropy, which accounts for the effect of recombination rate. Thus, it can be concluded that more pleiotropic variants are associated with stronger purifying selection. Therefore, even though highly pleiotropic loci detected by GWAS seem to have larger effect sizes (Chesmore et al. 2018 and our Fig. 1a) and frequencies (Fig. 1c), they seem to be subjected to stronger selection than less pleiotropic ones. Variants with a large effect size and a common frequency are easier to detect by GWAS (see Supplementary Material Table S3 for an illustration of this), so this may explain the observations. In fact, the magnitude of pleiotropy is inevitably underestimated because of sampling error and lack of power (Hill and Zhang 2012). In addition, the effect sizes refer to a quantitative trait that may be related with fitness to a higher or lower degree (Keightley and Hill 1990). It has been shown theoretically that variants with a large effect on a quantitative trait but a low correlated effect on fitness can be those more easily detected by GWAS and also those contributing more to the heritability of the trait (Caballero et al. 2015). Finally, in regions of low recombination, a reduction of the effective population size is expected (Hudson and Kaplan 1995; Nordborg et al. 1996; Santiago and Caballero 1998, 2016; Nicolaisen and Desai 2013; Caballero 2020, p. 106). This would imply a larger impact of genetic drift, and therefore, the possibility that deleterious alleles can reach higher frequencies than expected, as has been already shown for schizophrenia variants (Pardiñas et al. 2018). In summary, our results show that highly pleiotropic variants are associated with intense background selection, but those found by GWAS tend to have a larger effect and frequency than less pleiotropic variants. Thus, it may be hypothesized that an unknown number of highly pleiotropic variants of low effect/frequency may pass undetected by GWAS, explaining these results.

The study by Shikov et al. (2020) disclosed that protein-level pleiotropy due to ubiquitously expressed genes is the most prevalent form of pleiotropy. This is coherent with the recognized implication of the general metabolic pathways in pleiotropic effects (Kacser and Burns 1981). It is then consistent with the view that ubiquitous and general function proteins must be constrained by purifying selection. Note, however, that the *B* statistic can also be affected by other selection effects such as hitchhiking of favorable alleles and biased gene conversion (McVicker et al. 2009), so that its value does not only describe negative selection. In addition, many pleiotropic effects are expected to act in the same direction of reducing fitness, but some can operate as antagonistic pleiotropy (Rodríguez et al. 2017), as found for psychiatric disorders (Muntané et al. 2021). Thus, it is necessary to further disentangle the selection forces involved in highly pleiotropic loci.

## Supplementary Information

Below is the link to the electronic supplementary material.

Supplementary file1 (DOCX 1481 KB)

## Data Availability

Not applicable.

## Code availability

Computer codes and scripts will be available at Github address https://github.com/irene-novo-g/Pleiotropy.
