# Supplementary material for: Highly pleiotropic variants of human traits are enriched in genomic regions with strong background selection
Source: Hum Genet. 2021 Jul 6;140(9):1343–51. doi: 10.1007/s00439-021-02308-w (PMC8338839; doi:10.1007/s00439-021-02308-w)
Supplement: Supplementary file 1 — Supplementary file1 (DOCX 1481 KB) [file 439_2021_2308_MOESM1_ESM.docx]

**Highly pleiotropic variants of human traits are enriched in genomic regions with strong background selection**

Irene Novo, Eugenio López-Cortegano and Armando Caballero

Corresponding author: Irene Novo, Centro de Investigación Mariña, Universidade de Vigo, Departamento de Bioquímica, Xenética e Inmunoloxía, Facultade de Bioloxía, Edificio CC Experimentais, Campus de Vigo, 36310 Vigo, Spain. Tel: +34 986813828. Fax: +34 986812556. Email: irene.novo.gimenez@uvigo.es

**Table S1.** Number of loci analyzed in this study and pleiotropic loci found for each of 41 diseases and other human traits, grouped by functional domain.

| **Functional domain** | **Trait** | **Loci** | **Pleio loci** |
| --- | --- | --- | --- |
| Cancer | Basal cell carcinoma | 37 | 15 |
|  | Chronic lymphocytic leukemia | 35 | 19 |
|  | Lung cancer | 66 | 20 |
|  | Prostate cancer | 95 | 32 |
|  | Prostate-specific antigen levels | 28 | 11 |
|  | Testicular germ cell tumor | 50 | 7 |
| Cardiovascular | Atrial fibrillation | 27 | 4 |
|  | Coronary artery disease | 43 | 29 |
|  | Coronary heart disease | 35 | 28 |
|  | Myocardial infarction | 29 | 23 |
| Dermatological | Atopic dermatitis | 25 | 5 |
|  | Psoriasis | 47 | 16 |
|  | Vitiligo | 51 | 26 |
| Endocrine | Body mass index | 270 | 110 |
|  | Menarche (age at onset) | 103 | 27 |
|  | Obesity | 37 | 32 |
|  | Type 2 diabetes | 81 | 40 |
| Gastrointestinal | Digestive disease | 248 | 121 |
|  | Ulcerative colitis | 107 | 81 |
| Hematological | Glycated hemoglobin levels | 51 | 22 |
|  | Mean platelet volume | 229 | 48 |
|  | Monocyte count | 193 | 88 |
|  | Neutrophil traits | 237 | 102 |
|  | Red blood cell traits | 42 | 23 |
| Immunological | Primary biliary cholangitis | 47 | 23 |
|  | Rheumatoid arthritis | 92 | 43 |
|  | Systemic lupus erythematosus | 75 | 35 |
|  | Type 1 diabetes | 36 | 18 |
| Metabolic | Cholesterol | 149 | 68 |
|  | HDL | 125 | 64 |
|  | Triglycerides | 78 | 51 |
|  | Urate levels | 34 | 11 |
| Neurological / Psychiatric | Migraine | 36 | 8 |
|  | Parkinson's disease | 58 | 8 |
|  | Schizophrenia | 107 | 20 |
| Skeletal | Bone mineral density | 54 | 11 |
|  | Height | 322 | 98 |
|  | Waist circumference | 49 | 42 |
|  | Waist-related traits | 121 | 92 |
|  | Waist-hip ratio | 41 | 35 |
|  | Waist-to-hip related traits | 81 | 53 |

**Table S2.** List of the most pleiotropic known genes or inter-genic regions found in this study, indicating the chromosome, start and end of gene location, the pleiotropic degree and the value of the average background statistic *B* for the whole length of the gene.

| **Gene** | **Full name** | **Ch.** | **Location** | **Pleio** | ***B*** |
| --- | --- | --- | --- | --- | --- |
| CDKN2B-AS1 - DMRTA1 | CDKN2B antisense RNA 1 - DMRT like family A1 | 9 | 21994790  22455739 | 10 | 0.918062 |
| FTO | FTO alpha-ketoglutarate dependent dioxygenase | 16 | 53737875  54155853 | 9 | 0.867486 |
| HLA-DRB9 - HLA-DRB5 | major histocompatibility complex, class II, DR beta 9 - DR beta 5 | 6 | 32427597 32498064 | 8 | 0.927388 |
| PPARG | peroxisome proliferator activated receptor gamma | 3 | 12328867  12475843 | 6 | 0.743163 |
| FADS1 | fatty acid desaturase 1 | 11 | 61567099  61584475 | 6 | 0.498626 |
| GCKR | glucokinase regulator | 2 | 27719706  27746551 | 6 | 0.099403 |
| LPA | lipoprotein(a) | 6 | 160952514  161085307 | 6 | 0.790012 |
|  |  |  |  |  |  |
| AP4B1-AS1 PTPN22 | AP4B1 antisense RNA 1 - protein tyrosine phosphatase non-receptor type 22 | 1 | 114355234 114414381 | 6 | 0.126539 |
| ATXN2 | ataxin 2 | 12 | 111890018  112037477 | 6 | 0.116961 |
| HLA-DQB1 - MTCO3P1 | major histocompatibility complex, class II, DQ beta 1 - MT-CO3 pseudogene 1 | 6 | 32627244 32674580 | 7 | 0.940000 |
| JAZF1 | JAZF zinc finger 1 | 7 | 27870196  28220414 | 8 | 0.809438 |
| TYK2 | tyrosine kinase 2 | 19 | 10461209  10491248 | 7 | 0.705571 |
| PEPD | peptidase D | 19 | 33877856  34012697 | 6 | 0.829497 |

**Table S3.** Associations of the intergenic region CDKN2B-AS1 - DMRTA1 with multiple traits (pleiotropy degree = 10). Columns indicate the trait affected, effect (*β*), minor allele frequency (MAF), sample size (*n*), power of a statistical test to detect association by GWAS estimated by the non-centrality parameter (NCP) using Equation A4 of Visscher et al. (2017), and corresponding power if the effect is halved. NCP is obtained as NCP = *n* × MAF × (1 – MAF) × *β*^2^, assuming that there is a full correlation between the genotyped SNP and the causal variant. The average power of detection per trait is reduced from 47 % to 20 %.

| Trait | *β* | MAF | *n* | NCP (%) | NCP (%) for ½ *β* |
| --- | --- | --- | --- | --- | --- |
| Myocardial infarction | 0.066 | 0.45 | 17,624 | 38.4 | 9.6 |
| Type 2 diabetes | 0.079 | 0.45 | 12,999 | 40.4 | 10.1 |
| Coronary heart disease | 0.067 | 0.49 | 59,789 | 100 | 33.8 |
| Lung cáncer | 0.030 | 0.07 | 26078 | 3.2 | 0.8 |
| Atopic dermatitis | 0.062 | 0.49 | 9,274 | 17.8 | 4.4 |
| Chronic lymphocytic leukemia | 0.031 | 0.50 | 18,255 | 9.0 | 2.3 |
| Monocyte count | 0.046 | 0.16 | 170,721 | 99.1 | 24.8 |
| Neutrophil traits | 0.038 | 0.11 | 172,435 | 50.1 | 12.7 |
| Glycated hemoglobin levels | 0.014 | 0.20 | 158,869 | 10.3 | 2.6 |
| Body mass index | 0.320 | 0.42 | 495,584 | 100 | 100 |
| Average | 0.075 | 0.33 | 114,163 | 46.8 | 20.1 |

**Figure S1.** Proportion of pleiotropic genes found in this study for each of 41 traits grouped in functional domains.


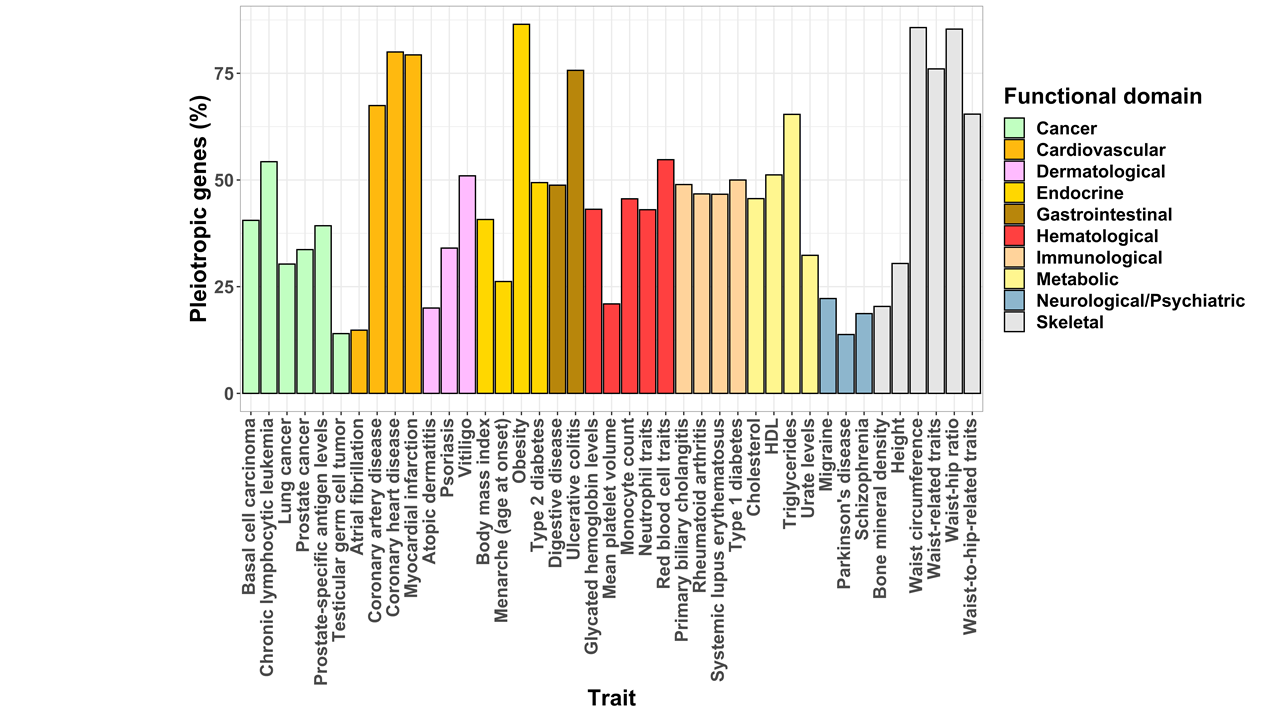


**Figure S2**. Proportion of genes found in this study for each degree of pleiotropy.


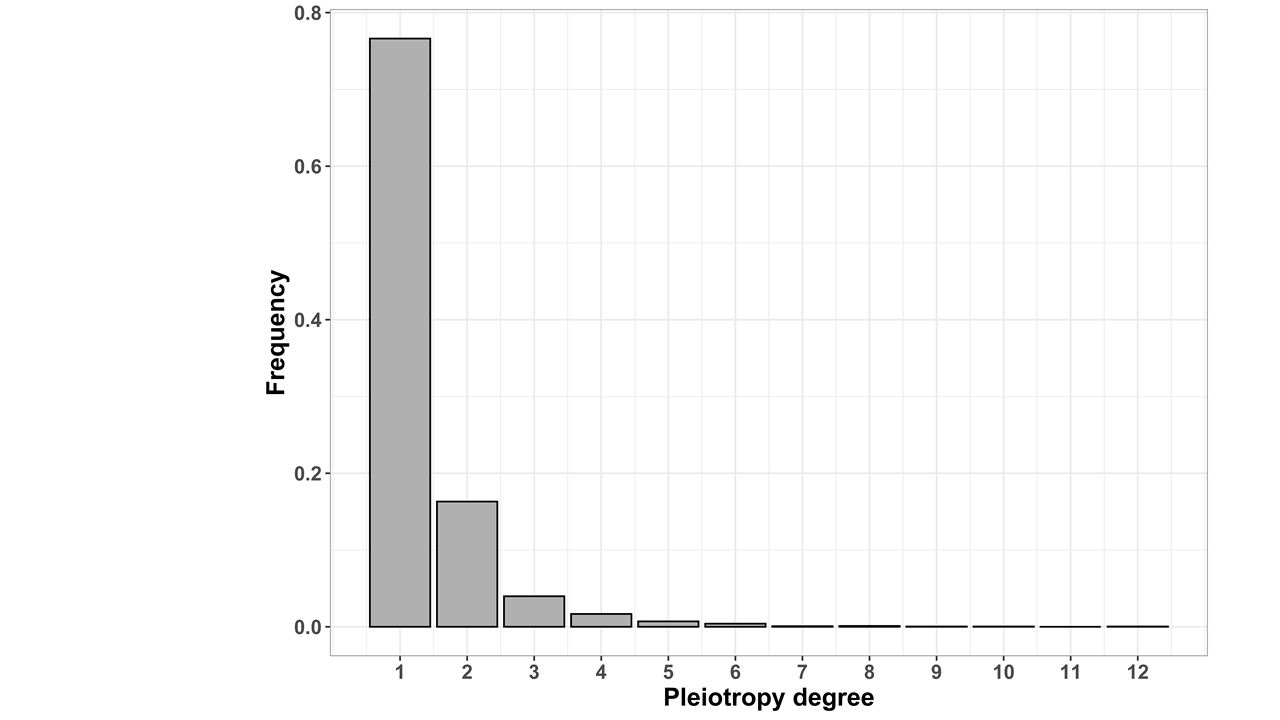


**Figure S3.** Relationship between the background selection statistic (*B*) and the degree of pleiotropy of data found by Watanabe et al. (2019). (a) Average *B* value for genomic regions considering pleiotropy across traits (simple regression *b* = –0.002, R^2^ = 0.09, F = 321, *p* < 2 × 10^–16^; partial regression *b´* = –0.003, R^2^ = 0.24, F = 521.4, *p* < 2 × 10^–16^). (b) Average *B* value for genomic regions considering pleiotropy across domains (simple regression *b* = –0.015, R^2^ = 0.09, F = 327.7, *p* < 2 × 10^–16^; partial regression *b´* = –0.018, R^2^ = 0.24, F = 534, *p* < 2 × 10^–16^) (presented also in Figure 3c of the main text). (c) Average *B* value for genes considering traits (simple regression *b* = –0.005, R^2^ = 0.06, F = 723.3, *p* < 2 × 10^–16^; partial regression *b´* = –0.003, R^2^ = 0.39, F = 3606, *p* < 2 × 10^–16^); (d) Average *B* value for genes considering pleiotropy across domains (simple regression *b* = –0.035, R^2^ = 0.09, F = 1148, *p* < 2 × 10^–16^; partial regression *b´* = –0.024, R^2^ = 0.40, F = 3815, *p* < 2 × 10^–16^); (e) Average *B* value for SNPs considering traits (simple regression *b* = –0.007, R^2^ = 0.03, F = 8412, *p* < 2 × 10^–16^; partial regression *b´* = –0.005, R^2^ = 0.15, F = 21300, *p* < 2 × 10^–16^); (f) Average *B* value for SNPs considering pleiotropy across domains (simple regression *b* = –0.143, R^2^ = 0.02, F = 4602, *p* < 2 × 10^–16^; partial regression *b´* = –0.046, R^2^ = 0.13, F = 17930, *p* < 2 × 10^–16^). Simple regression lines are shown.


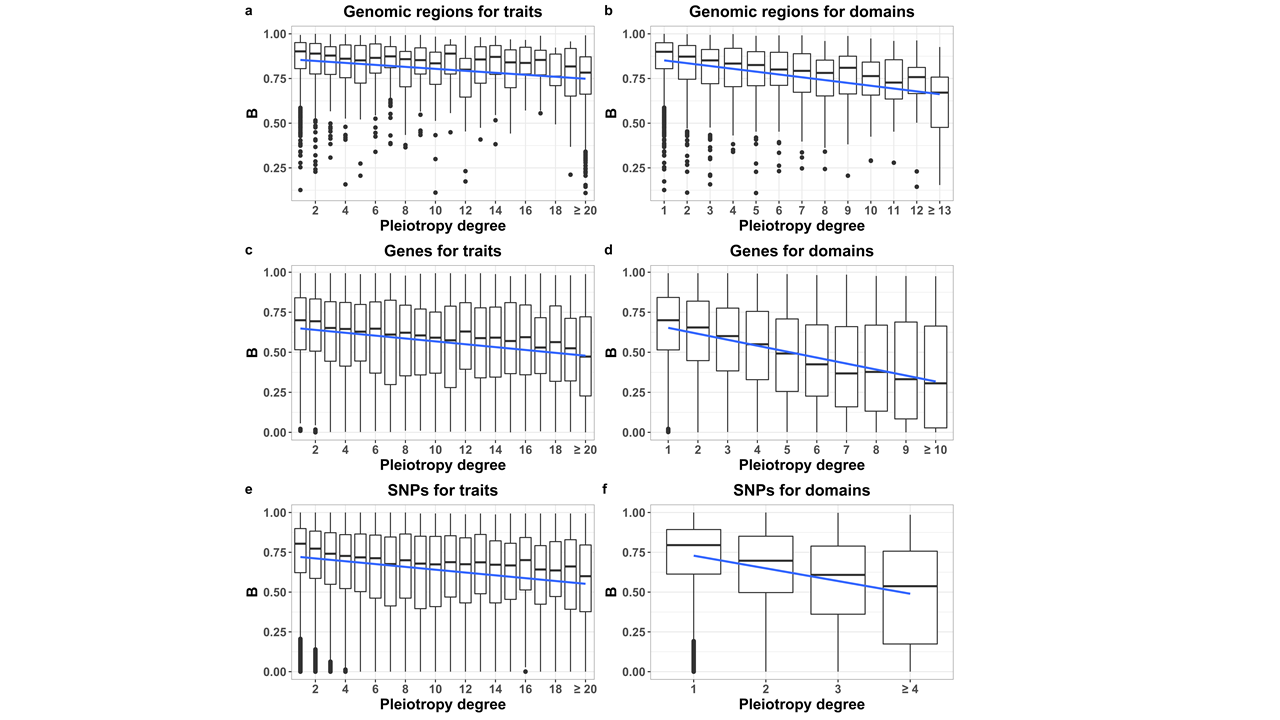


**Figure S4.** Relationship between the background selection statistic (*B*) and the degree of pleiotropy of data found by Shikov et al. (2020). (a) Average *B* value for genomic regions considering the median degree of pleiotropy for each region (simple regression *b* = –0.026, R^2^ = 0.06, F = 83.14, *p* < 2 × 10^–16^; partial regression *b´* = –0.021, R^2^ = 0.38, F = 399.2, *p* < 2 × 10^–16^). (b) Average *B* value for genomic regions considering the maximal degree of pleiotropy for each region (simple regression *b* = –0.026, R^2^ = 0.06, F = 87.02, *p* < 2 × 10^–16^; partial regression *b´* = –0.022, R^2^ = 0.38, F = 400.5, *p* < 2 × 10^–16^) (presented also in Figure 3d of the main text). (c) Average *B* value for non-MHC SNPs considering the 44,851 high confidence pleiotropic SNPs (simple regression *b* = –0.019, R^2^ = –0.002, F = 96.34, *p* < 2 × 10^–16^; partial regression *b´* = –0.008, R^2^ = 0.24, F = 7065, *p* = 3 × 10^–6^); (d) Average *B* value for non-MHC SNPs considering all 125,425 pleiotropic SNPs found (simple regression *b* = –0.002, R^2^ = 6 × 10^–6^, F = 1.72, *p* = 0.2; partial regression *b´* = –0.015, R^2^ = 0.18, F = 14,130, *p* < 2 × 10^–16^). Simple regression lines are shown.


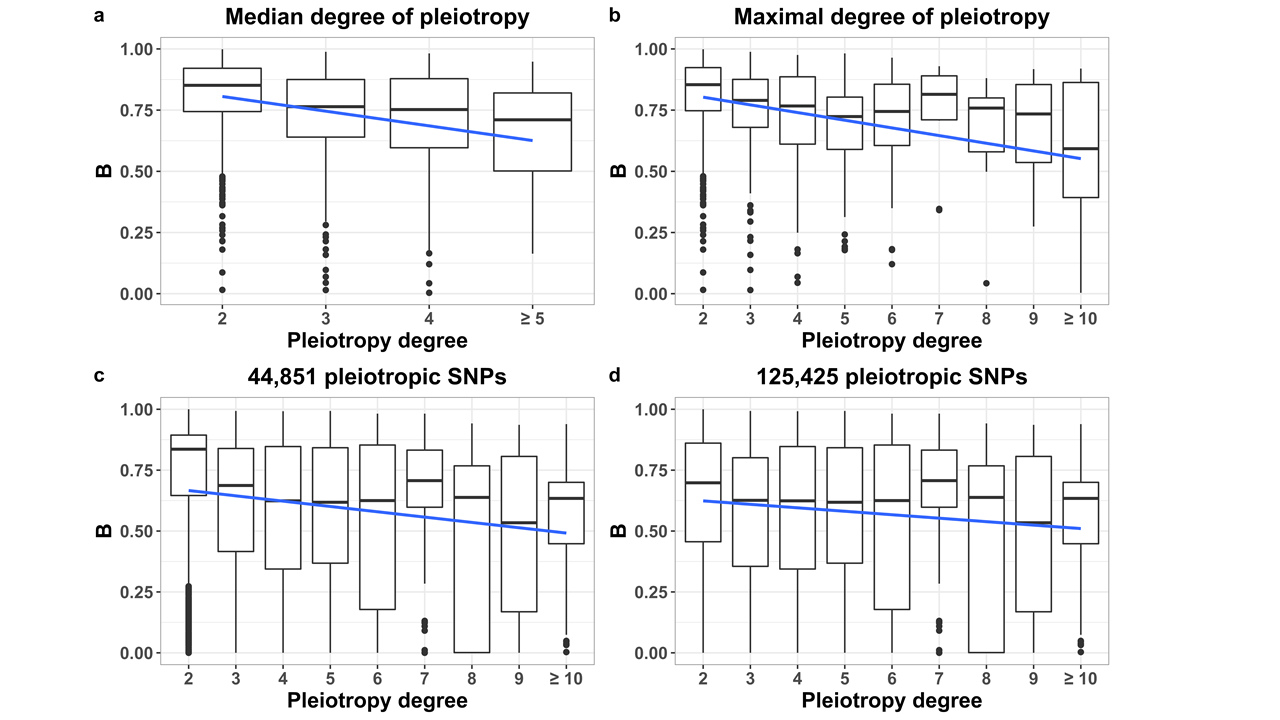


**Figure S5.** Relationship between the standard deviation of the average effect sizes of pleiotropic variants and the degree of pleiotropy (*b* = –0.008, R^2^ = 0.51, F = 11.23, *p* = 0.008).


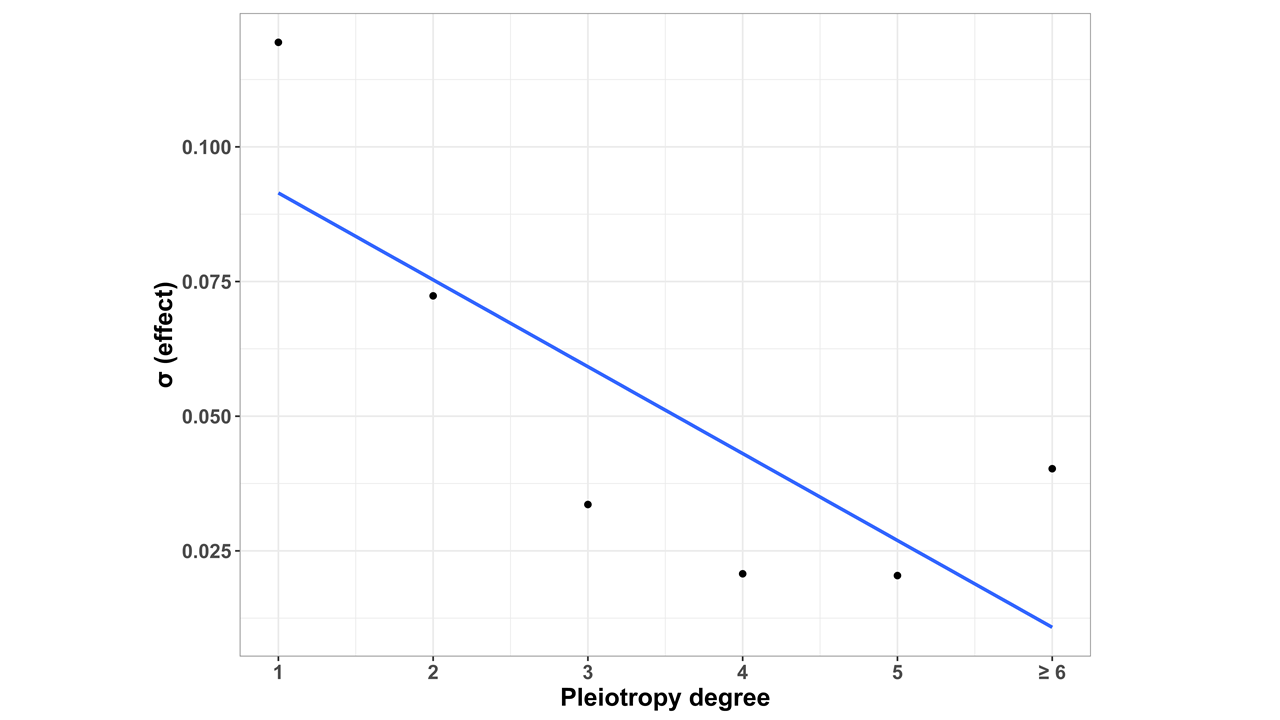


**Figure S6.** Average value of the *B* statistic for different degrees of pleiotropy for the different datasets analyzed. Averages of *B* values for genomic regions are shown by continuous lines, averages for genes by broken lines, and averages for SNPs by dotted lines. The last dot for each line includes data from that degree of pleiotropy or a larger one.

**
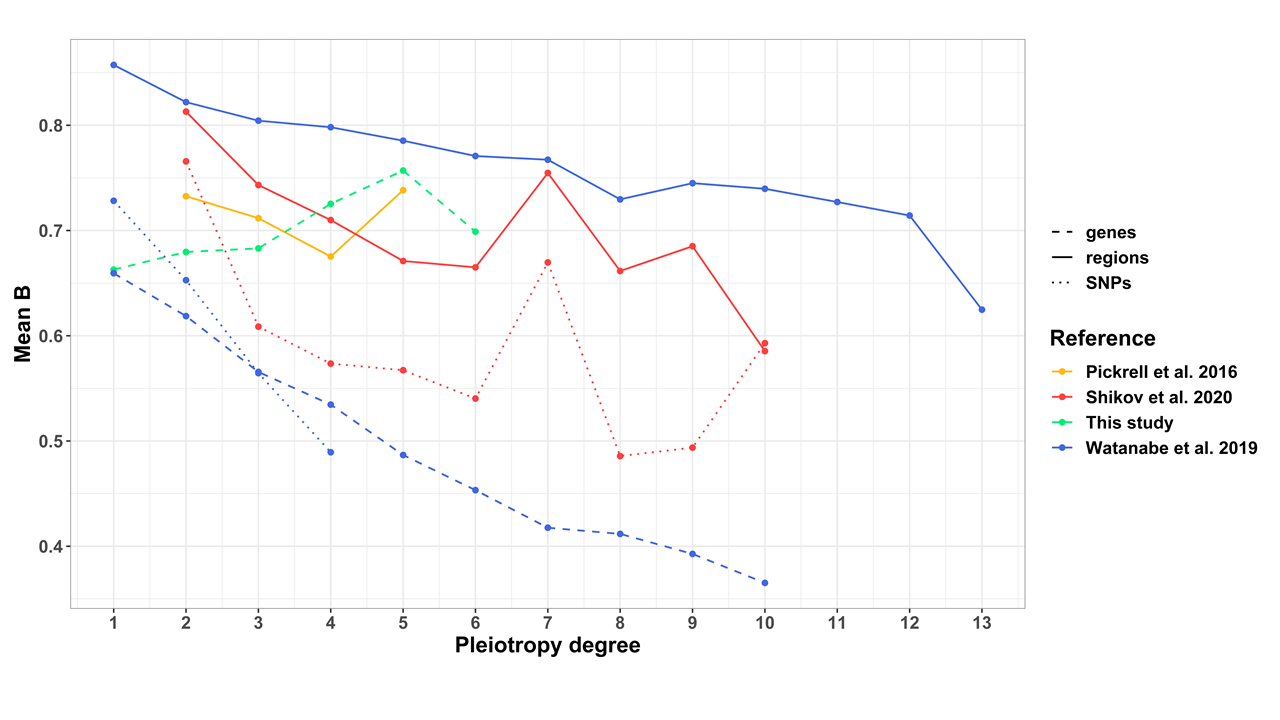
**
